# Avelumab (anti–PD-L1) as first-line switch-maintenance or second-line therapy in patients with advanced gastric or gastroesophageal junction cancer: phase 1b results from the JAVELIN Solid Tumor trial

**DOI:** 10.1186/s40425-019-0508-1

**Published:** 2019-02-04

**Authors:** Hyun Cheol Chung, Hendrik-Tobias Arkenau, Jeeyun Lee, Sun Young Rha, Do-Youn Oh, Lucjan Wyrwicz, Yoon-Koo Kang, Keun-Wook Lee, Jeffrey R. Infante, Sung Sook Lee, Margaret Kemeny, Ulrich Keilholz, Bohuslav Melichar, Alain Mita, Ruth Plummer, Denis Smith, Arnold B. Gelb, Huiling Xiong, Janet Hong, Vikram Chand, Howard Safran

**Affiliations:** 1 Yonsei Cancer Center, Yonsei University College of Medicine, Yonsei University Health System, Seoul, 03722 South Korea; 2 Sarah Cannon Research Institute/University College London, London, UK; 3 Samsung Medical Center, Sungkyunkwan University School of Medicine, Seoul, South Korea; 4 Seoul National University Hospital, Cancer Research Institute, Seoul National University College of Medicine, Seoul, South Korea; 5 Centrum Onkologii-Instytut im. M. Sklodowskiej Curie, Warszawa, Poland; 6 Asan Medical Center, University of Ulsan College of Medicine, Seoul, South Korea; 7 Seoul National University Bundang Hospital, Seoul National University College of Medicine, Seongnam, South Korea; 8 Sarah Cannon Research Institute/Tennessee Oncology, PLLC, Nashville, USA; 9 Inje University College of Medicine, Busan, South Korea; 10 Queens Cancer Center, Mt Sinai School of Medicine, New York, USA; 11 Charité Comprehensive Cancer Center, Charitéplatz 1, Berlin, Germany; 12 Palacky University Medical School and Teaching Hospital, I.P. Pavlova 6, Olomouc, Czech Republic; 13 Samuel Oschin Comprehensive Cancer Institute, Cedars-Sinai Medical Center, Los Angeles, USA; 14 Northern Centre for Cancer Care and Newcastle University, Freeman Hospital, Newcastle upon Tyne, UK; 15 Medical Oncology, Bordeaux University Hospital, Bordeaux CEDEX, France; 16 EMD Serono, Inc, Billerica, USA; 17 Brown University, Providence, USA; 18 Present address: AstraZeneca Pharmaceuticals LP, Gaithersburg, USA

**Keywords:** Avelumab, Metastatic, Gastric, Esophagogastric junction, Adenocarcinoma, Maintenance

## Abstract

**Background:**

We evaluated the antitumor activity and safety of avelumab, a human anti–PD-L1 IgG1 antibody, as first-line switch-maintenance (1 L-mn) or second-line (2 L) treatment in patients with advanced gastric/gastroesophageal cancer (GC/GEJC) previously treated with chemotherapy.

**Methods:**

In a phase 1b expansion cohort, patients without (1 L-mn) or with (2 L) disease progression following first-line chemotherapy for advanced GC/GEJC received avelumab 10 mg/kg intravenously every 2 weeks. Endpoints included best overall response, progression-free survival (PFS), overall survival (OS), and safety.

**Results:**

Overall, 150 patients were enrolled (1 L-mn, *n* = 90; 2 L, *n* = 60) and median follow-up in the 1 L-mn and 2 L subgroups was 36.0 and 33.7 months, respectively. The confirmed objective response rate was 6.7% in both subgroups (95% CI, 2.5–13.9% and 1.8–16.2%, respectively), including complete responses in 2.2% of the 1 L-mn subgroup (*n* = 2). In the 1 L-mn and 2 L subgroups, median duration of response was 21.4 months (95% CI, 4.0–not estimable) and 3.5 months (95% CI, 2.8–8.3) and disease control rates were 56.7 and 28.3%, respectively. Median PFS in the 1 L-mn and 2 L subgroups was 2.8 months (95% CI, 2.3–4.1) and 1.4 months (95% CI, 1.3–1.5), with 6-month PFS rates of 23.0% (95% CI, 14.7–32.4%) and 7.9% (95% CI, 2.6–17.2%), and median OS was 11.1 months (95% CI, 8.9–13.7) and 6.6 months (95% CI, 5.4–9.4), respectively. In the 1 L-mn subgroup, median OS measured from start of 1 L chemotherapy was 18.7 months (95% CI, 15.4–20.6). Across both subgroups, 20.7% had an infusion-related reaction of any grade. Other common treatment-related adverse events (TRAEs) of any grade included fatigue (10.0%) and nausea (6.7%). Treatment-related serious adverse events occurred in 4.0% of patients. Overall, 8.7% had a grade ≥3 TRAE, including 1 treatment-related death.

**Conclusion:**

Avelumab showed clinical activity and an acceptable safety profile in patients with GC/GEJC.

**Trial registration:**

ClinicalTrials.gov NCT01772004; registered 21 January 2013.

**Electronic supplementary material:**

The online version of this article (10.1186/s40425-019-0508-1) contains supplementary material, which is available to authorized users.

## Background

Gastric cancer (GC) is an aggressive disease that represents the third leading cause of cancer-related death worldwide [1]. Gastroesophageal junction cancer (GEJC) has similar biology, prognosis, and treatment guidelines as GC [2, 3]. First-line (1 L) standard of care for advanced inoperable GC/GEJC is based on combination fluoropyrimidine and platinum treatment, with trastuzumab added for HER2+ tumors. Second-line (2 L) options include regimens based on irinotecan, taxanes, and/or ramucirumab [2, 3]. However, cytotoxic regimens are associated with cumulative toxicity that may restrict long-term treatment, resulting in limited duration of response and overall survival (OS). Maintenance therapy, ie, continued treatment with an agent administered in the 1 L induction regimen or sequential treatment with a different agent until progression (switch maintenance), has the potential to extend durations of response and OS, particularly when an agent with a different mechanism of action is employed, while avoiding potential additive toxicity associated with further chemotherapy or combination treatment. As such, maintenance therapy has become an established strategy for several advanced tumors [4, 5]. Although the role of maintenance therapy in treating GC/GEJC is less well defined, observational and retrospective studies of maintenance fluoropyrimidine treatment in advanced GC/GEJC have shown that this approach is feasible and may improve progression-free survival (PFS) compared with observation alone [6–8].

In recent years, much attention has been focused on anticancer therapies that activate the immune response. In a randomized phase 2 study of patients with advanced GC/GEJC, switch-maintenance ipilimumab (anti–CTLA-4) after 1 L chemotherapy did not improve immune-related PFS or OS compared with best supportive care, which included continued fluoropyrimidine chemotherapy in most patients [9]. PD-L1 is a key therapeutic target for reactivating antitumor immune responses [10]. Additionally, PD-L1 is expressed in ≈30 to 60% of GC/GEJC specimens, with a higher frequency seen in certain pathological and genomic subtypes [11]. Immunotherapy with anti–PD-1 antibodies has been associated with durable antitumor responses in early-phase studies of patients with GC/GEJC [10, 12, 13].

Avelumab is a human anti–PD-L1 monoclonal antibody that has been approved in various countries for the treatment of metastatic Merkel cell carcinoma and in the United States and Canada for the treatment of advanced urothelial carcinoma progressing after platinum-containing chemotherapy. In phase 1 and 2 studies across various advanced cancers, avelumab has demonstrated a tolerable safety profile and durable antitumor activity [14–16]. In preclinical studies, avelumab activated both adaptive and innate immune effector cells [17, 18], suggesting an additional mechanism of action compared with other approved anti–PD-1/PD-L1 antibodies.

To investigate the efficacy and safety of avelumab in the treatment of advanced GC/GEJC, we enrolled a cohort of patients in the phase 1 JAVELIN Solid Tumor trial. Patients were enrolled following 1 L chemotherapy; those without disease progression received avelumab as 1 L switch maintenance (1 L-mn subgroup), and those with disease progression received avelumab as 2 L treatment (2 L subgroup). To our knowledge, this is the first study of an anti–PD-L1 agent administered as switch-maintenance therapy in this disease.

## Methods

### Study design and patients

JAVELIN Solid Tumor (NCT01772004) is an international, open-label, phase 1 trial. In the phase 1b, nonrandomized expansion cohort reported here, eligible patients had histologically confirmed, unresectable, locally advanced or metastatic GC/GEJC, and previous treatment with 1 L combination chemotherapy; patients with prior neoadjuvant platinum-based doublet or triplet chemotherapy who were not candidates for surgery were also eligible. Patients should not have received >1 line of prior treatment for metastatic disease, and patients with prior checkpoint inhibitor or trastuzumab treatment were ineligible (Additional file 1: Table S1). Patients in the 2 L subgroup were not permitted to have received anticancer treatment within 28 days before the start of study treatment, whereas in the 1 L-mn subgroup, patients were permitted to be enrolled within 28 days if all toxicity from prior therapy had resolved to grade ≤1. A fresh or archival tumor specimen was required, but patients were not preselected based on PD-L1 status (ie, all-comer design). Patients were enrolled in accordance with an approved protocol, international standards of good clinical practice, and institutional safety monitoring, and written informed consent was obtained. The study protocol was approved by the institutional review board or independent ethics committee at each center.

### Procedures and assessments

Patients received avelumab 10 mg/kg intravenously every 2 weeks until confirmed disease progression, unacceptable toxicity, or protocol-based criteria for withdrawal [15]. Premedication with diphenhydramine and acetaminophen was required 30 to 60 min before all avelumab infusions.

Safety was assessed at each biweekly trial visit and included assessment of adverse events (AEs), physical examination, clinical laboratory tests (hematology, hepatic panels, and serum chemistry), and documentation of concurrent medications. AEs and laboratory abnormalities were classified and graded according to National Cancer Institute Common Terminology Criteria for Adverse Events version 4.0. A serious AE (SAE) was defined as any untoward event that was life-threatening, required hospitalization, resulted in disability, was a congenital anomaly, or resulted in death. Immune-related AEs were identified using a prespecified list of Medical Dictionary for Regulatory Activities terms plus medical review. Clinical activity was assessed by investigators using Response Evaluation Criteria in Solid Tumors version 1.1. Radiographic tumor assessments were performed at baseline and every 6 weeks. In patients achieving a partial response (PR) or complete response (CR), a confirmatory CT or MRI scan was done ≥28 days later (preferably at the scheduled 6-week interval). PD-L1 expression was assessed in tumor cells using a proprietary immunohistochemistry assay (PD-L1 IHC 73-10; Dako, Carpinteria, CA), as described previously [15, 19]; in this report, PD-L1 status was defined using cutoffs of ≥1% of tumor cells positive for partial or complete membrane PD-L1 staining of any intensity. HER2 and microsatellite status were recorded retrospectively from medical records when available.

### Outcomes

Primary endpoints for the entire JAVELIN Solid Tumor trial were dose-limiting toxicities during the first 3 weeks of treatment in the phase 1a dose-escalation part (reported previously [20]) and confirmed best overall response adjudicated by independent review in specified efficacy expansion cohorts (not including the GC/GEJC cohort reported here). Secondary endpoints assessed in the current cohort included investigator-assessed best overall response, duration of response, PFS, OS, safety, and evaluation of PD-L1 expression [15].

### Statistical methods

Enrollment of 150 patients was planned for this cohort based on the anticipated sample size required to estimate and provide 95% Clopper-Pearson confidence intervals (CIs) for potential objective response rates (ORR; proportion of patients with a confirmed CR or PR; eg, 10% [5.7–16.0%] for 15 responders or 20% [13.9–27.3%] for 30 responders). Safety and antitumor activity were analyzed in all patients who received ≥1 dose of avelumab. Time-to-event endpoints (PFS, OS, duration of response, and duration of follow-up) were estimated using the Kaplan–Meier method and CIs for the median were calculated using the Brookmeyer–Crowley method.

## Results

### Patients

Between 13 February 2014 and 11 August 2015, 150 patients with histologically confirmed GC/GEJC were enrolled, including 90 without disease progression after 1 L chemotherapy (1 L-mn subgroup) and 60 with progressive disease (2 L subgroup), per investigator assessment (Table 1). In the 1 L-mn subgroup, 27.8% had achieved a PR with prior chemotherapy; in the 2 L subgroup, prior responses were PR in 13.3% and CR in 1.7%. The median interval between end of prior chemotherapy and start of avelumab was 45 days (1 L-mn subgroup) and 77 days (2 L subgroup). Across both subgroups, 30.7% had PD-L1+ tumors.

**Table 1:** Baseline characteristics in the first-line switch-maintenance and second-line subgroups

Characteristics	1 L-mn subgroup (*n* = 90)	2 L subgroup (*n* = 60)
Median age (IQR), years	59 (52.0–67.0)	62.5 (51.5–66.0)
Sex, n (%)		
Male	68 (75.6)	46 (76.7)
Female	22 (24.4)	14 (23.3)
ECOG PS, n (%)		
0	37 (41.1)	23 (38.3)
1	53 (58.9)	37 (61.7)
Geographic region, n (%)		
North America	31 (34.4)	32 (53.3)
Asia	34 (37.8)	10 (16.7)
Europe	25 (27.8)	18 (30.0)
Race, n (%)		
White	44 (48.9)	36 (60.0)
Asian	35 (38.9)	13 (21.7)
Black	4 (4.4)	4 (6.7)
Other	7 (7.8)	7 (11.7)
Histology, n (%)		
Tubular	18 (20.0)	3 (5.0)
Signet ring	17 (18.9)	13 (21.7)
Mucinous	4 (4.4)	4 (6.7)
Papillary	1 (1.1)	0
Other/not specified	1 (1.1)	0
Unknown	37 (41.1)	39 (65.0)
PD-L1 expression status based on ≥1% cutoff on tumor cells, n (%)		
PD-L1+	26 (28.9)	20 (33.3)
PD-L1−	51 (56.7)	25 (41.7)
Not evaluable	13 (14.4)	15 (25.0)
HER2 status, n (%)		
HER2−	62 (68.9)	29 (48.3)
HER2+	4 (4.4)	5 (8.3)
Unknown	24 (26.7)	26 (43.3)
Microsatellite status, n (%)		
Low	1 (1.1)	0
Stable	21 (23.3)	17 (28.3)
High	2 (2.2)	2 (3.3)
Unknown	66 (73.3)	41 (68.3)
Prior gastrectomy, n (%)	24 (26.7)	14 (23.3)
Metastatic disease status at study entry, n (%)		
M0	5 (5.6)	2 (3.3)
M1	85 (94.4)	58 (96.7)
Tumor size at baseline^a^		
Median (IQR), mm	33 (19–52)	44 (25–69.5)
Unknown, n (%)	1 (1.1)	1 (1.7)
Best response to prior anticancer therapy, n (%)		
Complete response	0	1 (1.7)
Partial response	25 (27.8)	8 (13.3)
Stable disease	59 (65.6)	23 (38.3)
Progressive disease	0	22 (36.7)
Not evaluable or unknown	6 (6.7)	5 (8.3)
Prior anticancer therapy (any setting), n (%)	90 (100)	60 (100)
Number of prior lines of anticancer therapy for metastatic or locally advanced disease, n (%)		
0	1 (1.1)	5 (8.3)
1	87 (96.7)	53 (88.3)
2	2 (2.2)	1 (1.7)
Unknown	0	1 (1.7)
Median prior lines (range)	1.0 (0–2)	1.0 (0–2)
Interval from end of prior chemotherapy to start of avelumab therapy		
Median (IQR), days	45 (35–64)	77 (49–135)
Data missing, n (%)	8 (8.9)	15 (25.0)

^a^Sum of the longest diameters of target lesions

Abbreviations: *1 L-mn* first-line switch-maintenance, *2 L* second line, *ECOG PS* Eastern Cooperative Oncology Group performance status, *IQR* interquartile range

At data cutoff (30 September 2017), patients in the 1 L-mn and 2 L subgroups had received a median (range) of 7 (1–79) and 4.5 (1–44) avelumab doses, and median duration of treatment was 3.2 months (interquartile range [IQR], 1.4–6.1) and 2.2 months (IQR, 1.4–5.2), respectively. Median duration of follow-up was 36.0 months (IQR, 33.7–37.7) in the 1 L-mn subgroup and 33.7 months (IQR, 27.9–34.9) in the 2 L subgroup. In both subgroups, the most common reason for treatment discontinuation was disease progression (1 L-mn, 75.6%; 2 L, 71.7%); other reasons were AE (13.3%, 10.0%), death (3.3%, 8.3%), withdrawal of consent (1.1%, 6.7%), loss to follow-up (0%, 1.7%), protocol noncompliance (1.1%, 0%), and physician decision (0%, 1.7%). Five patients remained on avelumab treatment at data cutoff, all in the 1 L-mn subgroup (5.6%).

### Antitumor activity: 1 L-mn subgroup

**Fig. 1:**
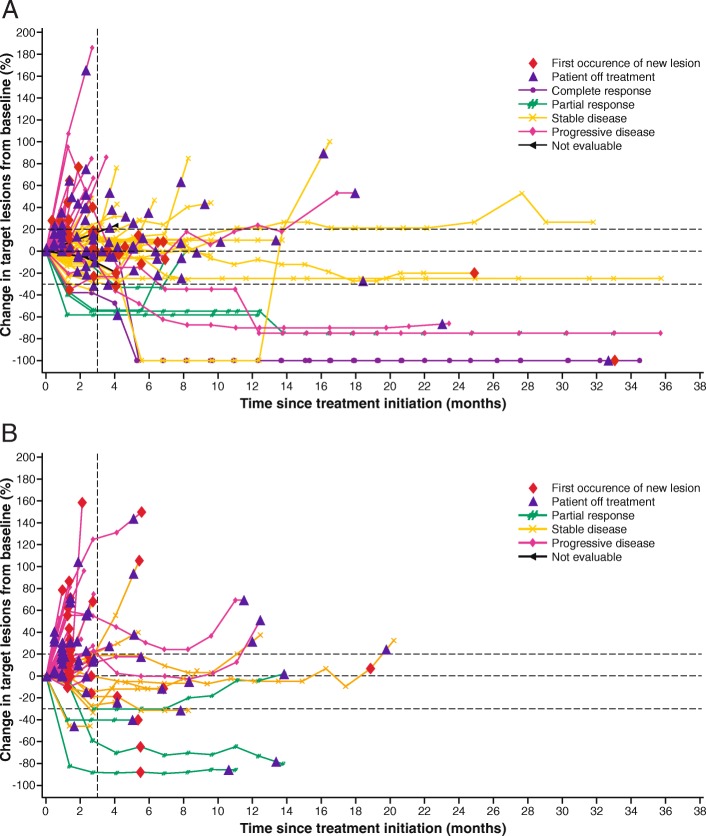
Change in sum of target lesion diameters over time with avelumab in evaluable patients. **a** First-line switch-maintenance subgroup (*n* = 81). **b** Second-line subgroup (*n* = 52). Color coding is based on best overall response per Response Evaluation Criteria in Solid Tumors version 1.1. Dotted lines indicate the 3-month timepoint and changes in target lesion size of −30, 0, and +20%

The confirmed ORR (additional effect after the end of chemotherapy) was 6.7% (*n* = 6; 95% CI, 2.5–13.9%) (Additional file 1: Table S2). Notably, 2 patients (2.2%) had a CR; both patients were Asian and had stable disease (SD) as best response to prior chemotherapy, and PD-L1 status was positive in 1 patient and not evaluable in the other. Four patients (4.4%) had a PR, which represented additional tumor shrinkage following prior chemotherapy; best response to prior chemotherapy in these patients was PR (*n* = 2) and SD (*n* = 2) (Additional file 1: Table S3). Forty-five patients (50.0%) had SD of any duration as best response (disease control rate, 56.7%). Early and durable responses were observed (Fig. 1a and Additional file 1: Figure S1A), with a median time to response of 1.4 months (IQR, 1.3–4.1), a median duration of response of 21.4 months (95% CI, 4.0–not estimable), and an estimated 66.7% (95% CI, 19.5–90.4%) of responses lasting ≥6 months. Responses were ongoing at data cutoff in 2 patients, including 1 patient with a CR. In evaluable patients with PD-L1+ or PD-L1− tumors, confirmed ORR was 7.7% (2/26; 95% CI, 0.9–25.1%) vs 3.9% (2/51; 95% CI, 0.5–13.5%). Of 81 patients evaluable for change in size of target lesions, 13 (16.0%) had shrinkage of ≥30% and 37 (45.7%) had shrinkage of any level (Additional file 1: Figure S2A). No correlation was seen between response to prior chemotherapy and tumor shrinkage on avelumab.

Two patients had prolonged shrinkage in target lesions with avelumab after documented progressive disease (due to >30% increase in target lesion size vs baseline at weeks 7–13 in 1 patient and a new lesion in the other patient), suggestive of pseudoprogression. Duration of avelumab treatment in these 2 patients was 36.2 months (ongoing) and 23.5 months, respectively. Another patient had SD before disease progression at week 13 (due to a new lesion), followed by a 100% reduction in target lesions sustained for >6 months.

**Fig. 2:**
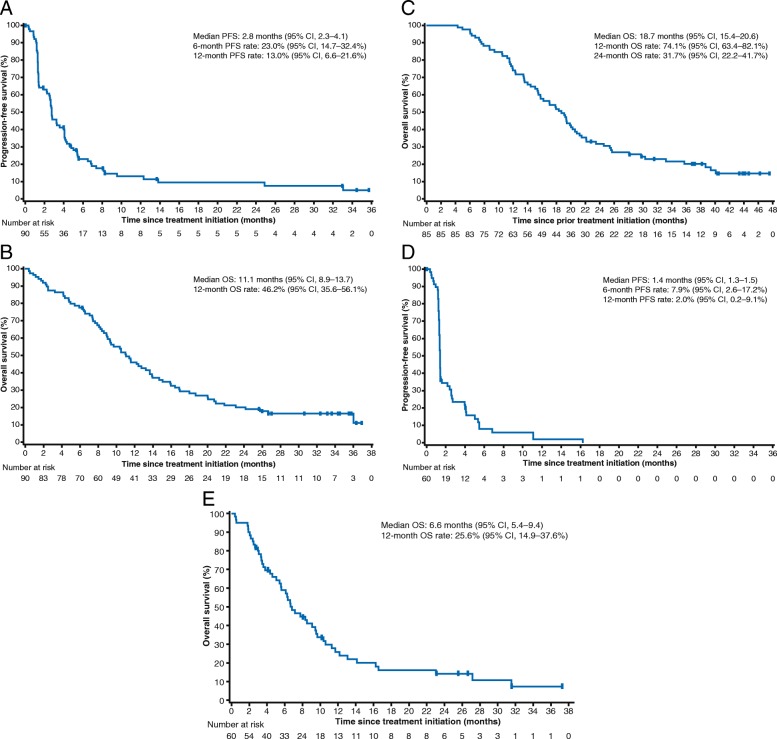
Kaplan–Meier estimates of progression-free survival (PFS) and overall survival (OS). **a** PFS from start of avelumab in the first-line switch-maintenance (1 L-mn) subgroup (*n* = 90). **b** OS from start of avelumab in the 1 L-mn subgroup (*n* = 90). **c** OS from start of 1 L chemotherapy in the 1 L-mn subgroup (*n* = 90). **d** PFS in the second-line (2 L) subgroup (*n* = 60). **e** OS in the 2 L subgroup (*n* = 60)

Median PFS and OS measured from start of avelumab therapy (ie, not including prior chemotherapy) were 2.8 months (95% CI, 2.3–4.1) and 11.1 months (95% CI, 8.9–13.7), respectively. The 6-month and 12-month PFS rates were 23.0% (95% CI, 14.7–32.4%) and 13.0% (95% CI, 6.6–21.6%), respectively, and the 12-month OS rate was 46.2% (95% CI, 35.6–56.1%) (Fig. 2a and b). In patients from Asian and non-Asian countries, median OS was 12.4 months (95% CI, 9.7–20.0) and 9.4 months (95% CI, 7.4–13.7), respectively. Median OS measured from start of 1 L chemotherapy was 18.7 months (95% CI, 15.4–20.6) overall (Fig. 2c), and 20.6 months (95% CI, 17.1–28.1) and 15.8 months (95% CI, 12.3–19.9) in patients from Asian and non-Asian countries, respectively. In PD-L1+ and PD-L1− subgroups, median PFS was 3.0 (95% CI, 1.4–4.1) and 2.7 (95% CI, 1.4–3.6) months (hazard ratio [HR], 0.844 [95% CI, 0.505–1.411]) and median OS was 15.9 (95% CI, 11.4–20.7) and 10.4 (95% CI, 8.3–12.4) months (HR, 0.588 [95% CI, 0.342–1.009]), respectively (Additional file 1: Figures S3A and S4A).

### Antitumor activity: 2 L subgroup

The ORR was 6.7% (95% CI, 1.8–16.2%; PR in 4 patients), and the disease control rate was 28.3% (13 patients [21.7%] had SD as best response) (Additional file 1: Table S2). Median time to and duration of response were 2.0 months (IQR, 1.3–2.7) and 3.5 months (95% CI, 2.8–8.3), respectively. An estimated 25.0% (95% CI, 0.9–66.5%) of responses lasted ≥6 months (Fig. 1b and Additional file 1: Figure S1B). Of 52 evaluable patients, 7 (13.5%) had target lesion shrinkage of ≥30% and 16 (30.8%) had shrinkage of any level (Additional file 1: Figure S2B). Median PFS was 1.4 months (95% CI, 1.3–1.5), and 6-month and 12-month PFS rates were 7.9% (95% CI, 2.6–17.2%) and 2.0% (95% CI, 0.2–9.1%), respectively (Fig. 2d). Median OS was 6.6 months (95% CI, 5.4–9.4), and the 12-month OS rate was 25.6% (95% CI, 14.9–37.6%) (Fig. 2e). In patients from Asian and non-Asian countries, median OS was 8.3 months (95% CI, 2.0–10.4) and 6.3 months (95% CI, 4.3–9.4), respectively. No significant difference was seen in PFS and OS based on PD-L1 status (Additional file 1: Figures S3B and S4B).

### Safety

Across both subgroups, 85 of 150 patients (56.7%) had a treatment-related AE (TRAE) of any grade, including 57 of 90 (63.3%) in the 1 L-mn subgroup and 28 of 60 (46.7%) in the 2 L subgroup. Patterns of TRAEs were similar in both subgroups (Table 2 and Additional file 1: Table S4). Overall, infusion-related reactions and related symptoms occurred in 20.7%. Time to onset of infusion-related reaction was first or second infusion in 29 of 31 cases (93.5%). Other common TRAEs (any grade in ≥5%) were fatigue (10.0%), nausea (6.7%), chills (6.0%), and pruritus (5.3%). Thirteen patients (8.7%) had a grade ≥3 TRAE (1 L-mn subgroup, 8 [8.9%]; 2 L subgroup, 5 [8.3%]), most commonly fatigue, asthenia, anemia, and elevated lipase (*n* = 2 each; 1.3%). One patient (0.7%) had a grade 3 infusion-related reaction (1 L-mn subgroup). Two patients (both 2 L subgroup) had a grade 4 TRAE: elevated lipase (*n* = 1) and decreased platelet count (*n* = 1). Overall, 81 patients (54.0%) had a SAE, which was related to treatment in 6 patients (4.0%; 3 in each subgroup). One treatment-related death occurred (1 L-mn subgroup) in a patient with peritoneal metastases and ascites at study entry who developed grade 5 autoimmune hepatitis and hepatic failure. Twenty-three patients (15.3%) had an immune-related AE, including grade ≥3 in 3 patients (2.0%): colitis (grade 3, 1 L-mn), autoimmune hepatitis/hepatic failure (grade 5, 1 L-mn), and adrenal insufficiency (grade 3, 2 L). Avelumab was permanently discontinued following a TRAE in 8 patients (5.3% overall; 1 L-mn: 6 [6.7%]; 2 L: 2 [3.3%]), of whom 3 (2.0%) discontinued because of an infusion-related reaction.

**Table 2:** Any-grade TRAEs occurring in ≥10% of patients or grade ≥3 in any patient and infusion-related reactions in the first-line switch-maintenance or second-line subgroup

Patients, n (%)	1 L-mn subgroup (*n* = 90)				2 L subgroup (*n* = 60)			
	Any grade	Grade 3	Grade 4	Grade 5	Any grade	Grade 3	Grade 4	Grade 5
Any TRAE^a^	57 (63.3)	7 (7.8)	0	1 (1.1)	28 (46.7)	3 (5.0)	2 (3.3)	0
Fatigue	10 (11.1)	2 (2.2)	0	0	5 (8.3)	0	0	0
Decreased appetite	3 (3.3)	0	0	0	2 (3.3)	1 (1.7)	0	0
Asthenia	1 (1.1)	0	0	0	3 (5.0)	2 (3.3)	0	0
Colitis	2 (2.2)	1 (1.1)	0	0	0	0	0	0
Elevated amylase	2 (2.2)	0	0	0	1 (1.7)	1 (1.7)	0	0
Elevated lipase	2 (2.2)	1 (1.1)	0	0	1 (1.7)	0	1 (1.7)	0
Elevated γ-glutamyltransferase	2 (2.2)	1 (1.1)	0	0	0	0	0	0
Anemia	1 (1.1)	1 (1.1)	0	0	1 (1.7)	1 (1.7)	0	0
Decreased platelet count	1 (1.1)	1 (1.1)	0	0	1 (1.7)	0	1 (1.7)	0
Abdominal pain	1 (1.1)	1 (1.1)	0	0	0	0	0	0
Adrenal insufficiency	1 (1.1)	0	0	0	1 (1.7)	1 (1.7)	0	0
Autoimmune hepatitis^b^	1 (1.1)	0	0	1 (1.1)	0	0	0	0
Decreased hemoglobin	1 (1.1)	1 (1.1)	0	0	0	0	0	0
Hepatic failure^b^	1 (1.1)	0	0	1 (1.1)	0	0	0	0
Hyperglycemia	1 (1.1)	1 (1.1)	0	0	0	0	0	0
Hypokalemia	1 (1.1)	1 (1.1)	0	0	0	0	0	0
Peripheral motor neuropathy	1 (1.1)	1 (1.1)	0	0	0	0	0	0
Infusion-related reaction^c^	20 (22.2)	1 (1.0)	0	0	11 (18.3)	0	0	0

^a^The incidence of treatment-related infusion-related reaction based on the single MedDRA preferred term is not listed

^b^Occurred in the same patient

^c^Includes adverse events categorized as infusion-related reaction, drug hypersensitivity, or hypersensitivity reaction that occurred on the day of infusion or day after infusion, in addition to signs and symptoms of infusion-related reaction that occurred on the same day of infusion and resolved within 2 days (including adverse events classified by investigators as related or unrelated to treatment)

*1 L-mn* first-line switch-maintenance, *2 L* second line, *TRAE* treatment-related adverse event

## Discussion

In this single-arm phase 1b cohort of 150 patients with previously treated advanced GC/GEJC, avelumab showed evidence of durable antitumor activity as 1 L-mn and 2 L therapy. The ORR was 6.7% in both subgroups, although median durations of response were 21.4 months in the 1 L-mn subgroup and 3.5 months in the 2 L subgroup. Remarkably, 2 patients (2.2%) in the 1 L-mn subgroup had a CR after achieving only SD on prior chemotherapy. Avelumab showed a tolerable safety profile, including a low rate of grade ≥3 TRAEs (8.7%) and immune-related AEs (any grade, 15.3%; grade ≥3, 2.0%), similar to observations in other tumor types [21]. Detailed guidance for recognizing and managing immune-related AEs with this class of agents have been published by consensus groups [22, 23]. The incidence of TRAEs of any grade was higher in the 1 L-mn subgroup compared with the 2 L subgroup (63.3% vs 46.7%), which may be due to the longer treatment duration and shorter interval from end of prior chemotherapy to start of avelumab in the 1 L-mn subgroup, although the incidence of grade ≥3 TRAEs was similar in both subgroups (8.9% vs 8.3%, respectively).

Approximately 70% of patients achieve a response or SD with standard 1 L chemotherapy [24, 25]; however, duration of OS is usually short [2, 3]. In the 1 L-mn subgroup, median PFS was 2.8 months (6-month rate, 23.0%), median OS measured from the start of avelumab was 11.1 months (12-month rate, 46.2%), and median OS measured from the start of prior chemotherapy was 18.7 months. Thus, the OS seen in the 1 L-mn subgroup, which enrolled patients without disease progression following chemotherapy, is encouraging for this subgroup of patients. Administering immunotherapy sequentially after completion of 1 L chemotherapy may enhance the immunostimulatory effects of chemotherapy while reducing the toxicity that may result when anti–PD-1 antibodies are administered in combination with other agents (eg, chemotherapy or ipilimumab) [26, 27]. To further assess this strategy, a randomized phase 3 trial is comparing avelumab switch-maintenance treatment with continuation of 1 L platinum-based chemotherapy in patients with advanced GC/GEJC (JAVELIN Gastric 100; NCT02625610).

Several early-phase studies assessed anti–PD-1 monotherapy in patients with chemotherapy-treated (later-line) GC/GEJC outside of the maintenance setting [12, 13, 27, 28], and median PFS and OS reported in non–PD-L1–selected populations were 2.0 months and 5.5–6.2 months, respectively. Survival data for avelumab (anti–PD-L1) in the 2 L subgroup (median PFS and OS of 1.4 and 6.6 months, respectively) appear consistent with these studies. Subsequently, phase 3 trials assessing later-line treatment with anti–PD-1/PD-L1 monotherapy in advanced GC/GEJC were initiated. In a randomized phase 3 trial of nivolumab vs placebo as third-line or later treatment in Asian patients with GC/GEJC (*n* = 493), the ORR was 11.2% vs 0% (*P*<.0001), median PFS was 1.6 vs 1.5 months (*P*<.0001), and median OS was 5.3 vs 4.1 months (*P*<.0001), respectively [13]. However, to date no improvement in OS has been shown in studies comparing single-agent checkpoint inhibitors with chemotherapy, such as trials of 2 L pembrolizumab vs paclitaxel (KEYNOTE-061) [29] and third-line avelumab vs physician choice of chemotherapy (JAVELIN Gastric 300) [30]. Results from phase 3 trials assessing alternative anti–PD-1/PD-L1–based regimens in the 1 L setting, such as switch-maintenance (sequential) or combination (concurrent) approaches, are needed.

Available data indicate that the benefits seen with anti–PD-1/PD-L1 antibodies in GC/GEJC may be limited to a small proportion of patients. Thus, predictive biomarkers to identify subpopulations more likely to respond to immunotherapy are a focus of ongoing research [10]. In this study, clinical activity was seen both in PD-L1+ and PD-L1− tumors including similar ORR and PFS and a nonsignificant trend in the 1 L-mn subgroup for longer OS in PD-L1+ tumors. It should be noted that the PD-L1 assay used in this study differs from those used in studies of other approved anti–PD-1/PD-L1 antibodies. Also, in the present study, PD-L1 status was based solely on tumor cell expression, whereas in studies of pembrolizumab in patients with GC/GEJC, in which antitumor activity was associated with PD-L1 expression, PD-L1 status was determined based on expression on tumor or immune cells (combined positive score). In addition, responses to pembrolizumab in patients with GC/GEJC have been associated with microsatellite instability–high/mismatch repair–deficient status and Epstein-Barr virus status [12, 31]. In the current trial, microsatellite status was available for only a small number of patients and findings were inconclusive. Assessment of novel biomarkers is planned for future avelumab studies in GC/GEJC.

## Conclusion

The data in the present phase 1b study demonstrate that avelumab administered as maintenance therapy (after disease control with standard chemotherapy) has antitumor activity and acceptable safety in patients with advanced GC/GEJC, supporting further investigations of this treatment approach.

## Additional file

Additional file 1: **Table S1.** Eligibility criteria. **Table S2.** Response to avelumab in the 1 L-mn and 2 L subgroups. **Table S3.** Best response to avelumab compared with best response to prior anticancer therapy in the 1 L-mn subgroup. **Table S4.** Overall summary of safety. **Figure S1.** Time to and duration of response in responding patients. **Figure S2.** Best change in sum of target lesion diameters from baseline with avelumab in evaluable patients. **Figure S3.** Progression-free survival by PD-L1 expression status (≥1% tumor cell cutoff) in evaluable patients. **Figure S4.** Overall survival by PD-L1 expression status (≥1% tumor cell cutoff) in evaluable patients. (DOCX 794 kb)

## Abbreviations

1 L-mn: First-line switch-maintenance
2 L: Second-line
AE: Adverse event
CR: Complete response
ECOG PS: Eastern Cooperative Oncology Group performance status
GC: Gastric cancer
GEJC: Gastroesophageal junction cancer
HR: Hazard ratio
ORR: Objective response rate
OS: Overall survival
PFS: Progression-free survival
PR: Partial response
SAE: Serious adverse event
SD: Stable disease
TRAE: Treatment-related adverse event
