# Supplementary material for: Avelumab (anti–PD-L1) as first-line switch-maintenance or second-line therapy in patients with advanced gastric or gastroesophageal junction cancer: phase 1b results from the JAVELIN Solid Tumor trial
Source: J Immunother Cancer. 2019 Feb 4;7:30. doi: 10.1186/s40425-019-0508-1 (PMC6362598; doi:10.1186/s40425-019-0508-1)

**Additional files**

**Table S1** Eligibility criteria.

| **Inclusion criteria** |
| --- |
| - Age ≥18 years - Histologically confirmed, unresectable locally advanced or metastatic, gastric or gastroesophageal junction adenocarcinoma (patients with HER2+ tumors were eligible) - Received first-line chemotherapy combination in the metastatic setting with or without disease progression - Received ≤1 line of treatment for metastatic disease - Patients who had received any platinum-containing doublet or triplet as a neoadjuvant chemotherapy but were not candidates for surgery were also eligible - Measurable disease with ≥1 unidimensional measurable lesion based on Response Evaluation Criteria in Solid Tumors version 1.1 - Availability of tumor archival material or fresh biopsies (excluding bone biopsies) - Eastern Cooperative Oncology Group performance status of 0 or 1 - Estimated life expectancy of ≥3 months - Adequate hematologic function (white blood cells ≥2 × 10^9^/L, absolute neutrophil count ≥1.0 × 10^9^/L, lymphocyte count ≥0.5 × 10^9^/L) - Adequate hepatic function, defined by a total bilirubin level of ≤1.5 × ULN, aspartate aminotransferase level of ≤2.5 × ULN, and alanine aminotransferase level of ≤2.5 × ULN - Adequate renal function, defined by an estimated creatinine clearance of >30 mL/min according to the Cockcroft-Gault formula or measured 24-hour creatinine clearance (or local institutional standard method) - Use of highly effective contraception - Signed written informed consent |
| **Exclusion criteria** |
| - Prior therapy with any antibody/drug targeting T-cell coregulatory proteins (immune checkpoints), such as anti–PD-1, anti–PD-L1, or anti–CTLA-4 antibody - Prior therapy with trastuzumab - Concurrent anticancer therapy or major surgery within 28 days before start of trial treatment (except for patients with disease that had not progressed on first-line chemotherapy, provided all toxicity from prior therapy had resolved to grade ≤1), use of hormonal agents within 7 days of start of trial treatment, or any other concurrent investigational treatment; patients receiving immunosuppressive agents, such as steroids, were required to be tapered off these before initiation of the study treatment, except for patients with adrenal insufficiency, who were allowed to continue corticosteroids at physiological replacement dose (prednisone ≤10 mg daily) or steroids with no or minimal systemic effect (topical, inhalation) - Previous malignant disease other than gastric or gastroesophageal cancer within the last 5 years, except for basal or squamous cell carcinoma of the skin or cervical carcinoma in situ - Rapidly progressive disease (eg, tumor lysis syndrome) - Active or history of central nervous system metastases - Receipt of any organ transplant, including allogeneic stem cell transplant - Clinically significant illness, including infection (HIV, hepatitis B or C virus); active or history of autoimmune disease (other than diabetes mellitus type 1, vitiligo, psoriasis, hypothyroid disease, or hyperthyroid disease not requiring immunosuppressive treatment) or immunodeficiency; cardiovascular disease; or any other significant diseases, which, in the opinion of the investigator, might impair patient tolerance of trial treatment - Persisting toxicity of grade >1 related to prior therapy (except grade ≤2 sensory neuropathy) - Known severe hypersensitivity to monoclonal antibodies (grade ≥3), history of anaphylaxis, or uncontrolled asthma - Vaccination within 4 weeks of the first dose of avelumab, except for inactivated vaccines - Psychiatric condition impacting the understanding or rendering of informed consent - Pregnancy or lactation - Known alcohol or drug abuse - Legal incapacity or limited legal capacity |

ULN, upper limit of normal.

**Table S2** Response to avelumab in the 1L-mn and 2L subgroups (based on Response Evaluation Criteria in Solid Tumors version 1.1)

|  | **1L-mn subgroup (*n*=90)** | **2L subgroup (*n*=60)** |
| --- | --- | --- |
| Best overall response, n (%) |  |  |
| Complete response | 2 (2.2) | 0 |
| Partial response | 4 (4.4) | 4 (6.7) |
| Stable disease | 45 (50.0) | 13 (21.7) |
| Progressive disease | 29 (32.2) | 36 (60.0) |
| Nonevaluable | 10 (11.1) | 7 (11.7) |
| Objective response rate, % (95% CI) | 6.7 (2.5-13.9) | 6.7 (1.8-16.2) |
| Disease control rate, % | 56.7 | 28.3 |

Nonevaluable includes “missing” and “not assessable.” Disease control rate is defined as complete response, partial response, and stable disease.

1L-mn, first-line switch-maintenance; 2L, second line.

**Table S3** Best response to avelumab compared with best response to prior anticancer therapy in the 1L-mn subgroup (n=90)

|  | **Best response to avelumab, n (%)** | | | | | |
| --- | --- | --- | --- | --- | --- | --- |
| Best response to prior anticancer therapy | **CR** | **PR** | **SD** | **PD** | **NE** | **Overall** |
| CR | 0 | 0 | 0 | 0 | 0 | 0 |
| PR | 0 | 2 (2.2) | 11 (12.2) | 9 (10.0) | 3 (3.3) | 25 (27.8) |
| SD | 2 (2.2) | 2 (2.2) | 30 (33.3) | 19 (21.1) | 6 (6.7) | 59 (65.6) |
| PD | 0 | 0 | 0 | 0 | 0 | 0 |
| NE | 0 | 0 | 4 (4.4) | 1 (1.1) | 1 (1.1) | 6 (6.7) |
| Overall | 2 (2.2) | 4 (4.4) | 45 (50.0) | 29 (32.2) | 10 (11.1) | 90 (100) |

1L-mn, first-line switch-maintenance; CR, complete response; NE, nonevaluable; PD, progressive disease; PR, partial response; SD, stable disease.

**Table S4** Overall summary of safety

| Patients, n (%) | **Overall (*N*=150)** | **1L-mn  (*n*=90)** | **2L  (*n*=60)** |
| --- | --- | --- | --- |
| Any AE | 148 (98.7) | 88 (97.8) | 60 (100) |
| Any TRAE | 85 (56.7) | 57 (63.3) | 28 (46.7) |
| Any grade ≥3 AE | 102 (68.0) | 56 (62.2) | 46 (76.7) |
| Any grade ≥3 TRAE | 13 (8.7) | 8 (8.9) | 5 (8.3) |
| Any serious AE | 81 (54.0) | 44 (48.9) | 37 (61.7) |
| Any serious TRAE | 6 (4.0) | 3 (3.3) | 3 (5.0) |
| Any AE leading to permanent treatment discontinuation | 22 (14.7) | 14 (15.6) | 8 (13.3) |
| Any TRAE leading to permanent treatment discontinuation | 8 (5.3) | 6 (6.7) | 2 (3.3) |
| Any AE leading to death | 20 (13.3) | 10 (11.1) | 10 (16.7) |
| Any TRAE leading to death | 1 (0.7) | 1 (1.1) | 0 |
| Any immune-mediated AE | 23 (15.3) | 17 (18.9) | 6 (10.0) |
| Any infusion-related reaction | 31 (20.7) | 20 (22.2) | 11 (18.3) |
| Any treatment-related infusion-related reaction | 31 (20.7) | 20 (22.2) | 11 (18.3) |

AE, adverse event; TRAE, treatment-related adverse event.

**Figure S1** Time to and duration of response in responding patients. (A) First-line (1L) switch-maintenance subgroup (*n*=6). (B) Second-line subgroup (*n*=4).

A.


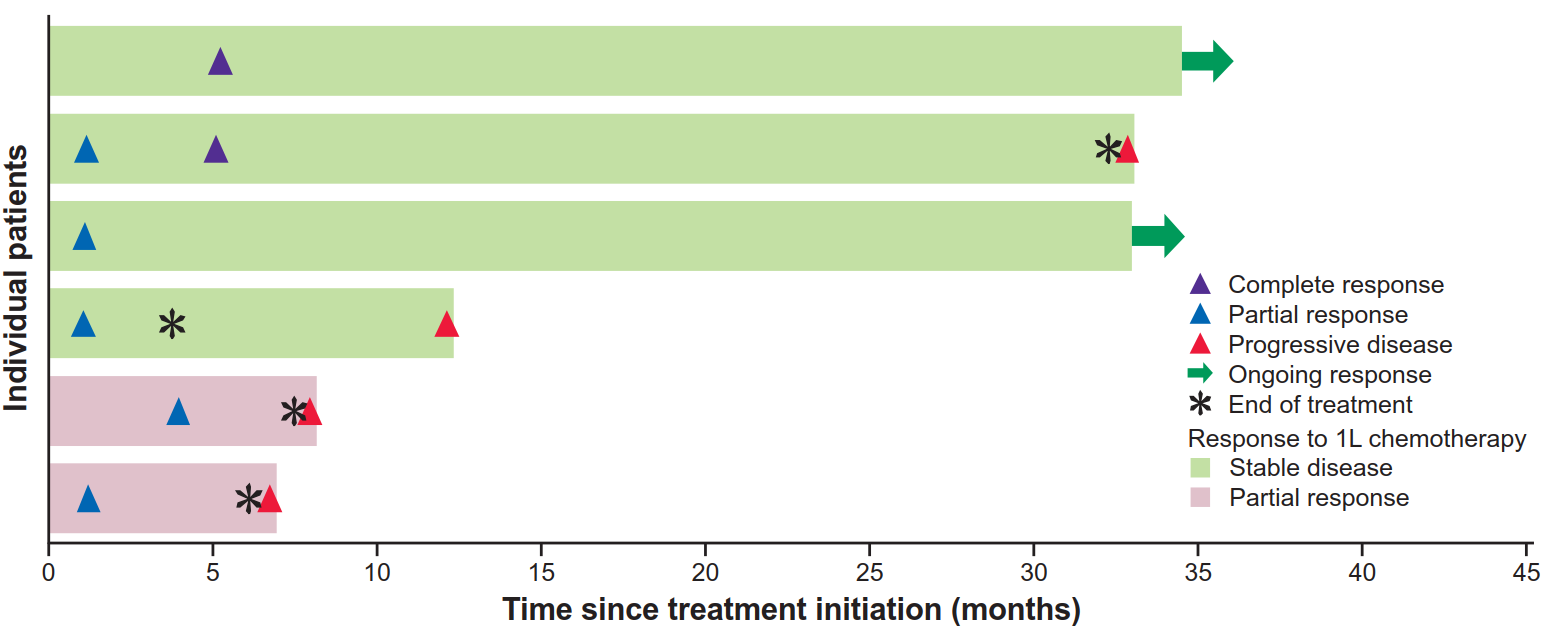


B.


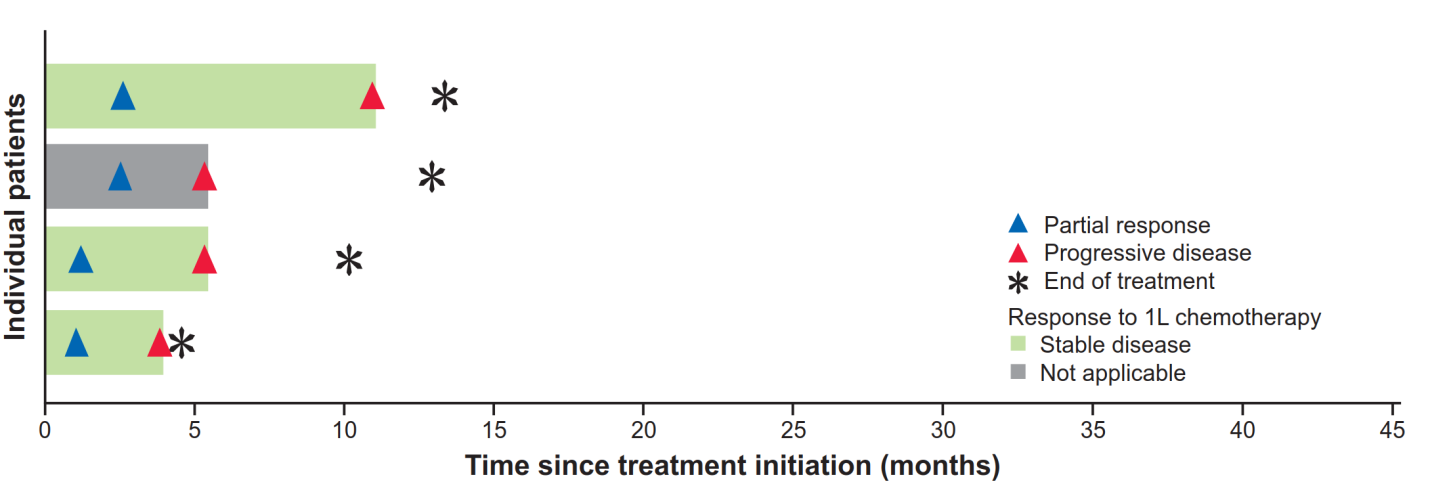


**Figure S2** Best change in sum of target lesion diameters from baseline with avelumab in evaluable patients, with color coding based on best overall response per Response Evaluation Criteria in Solid Tumors version 1.1. (A) First-line switch-maintenance subgroup (*n*=81). (B) Second-line subgroup (*n*=52).

A.


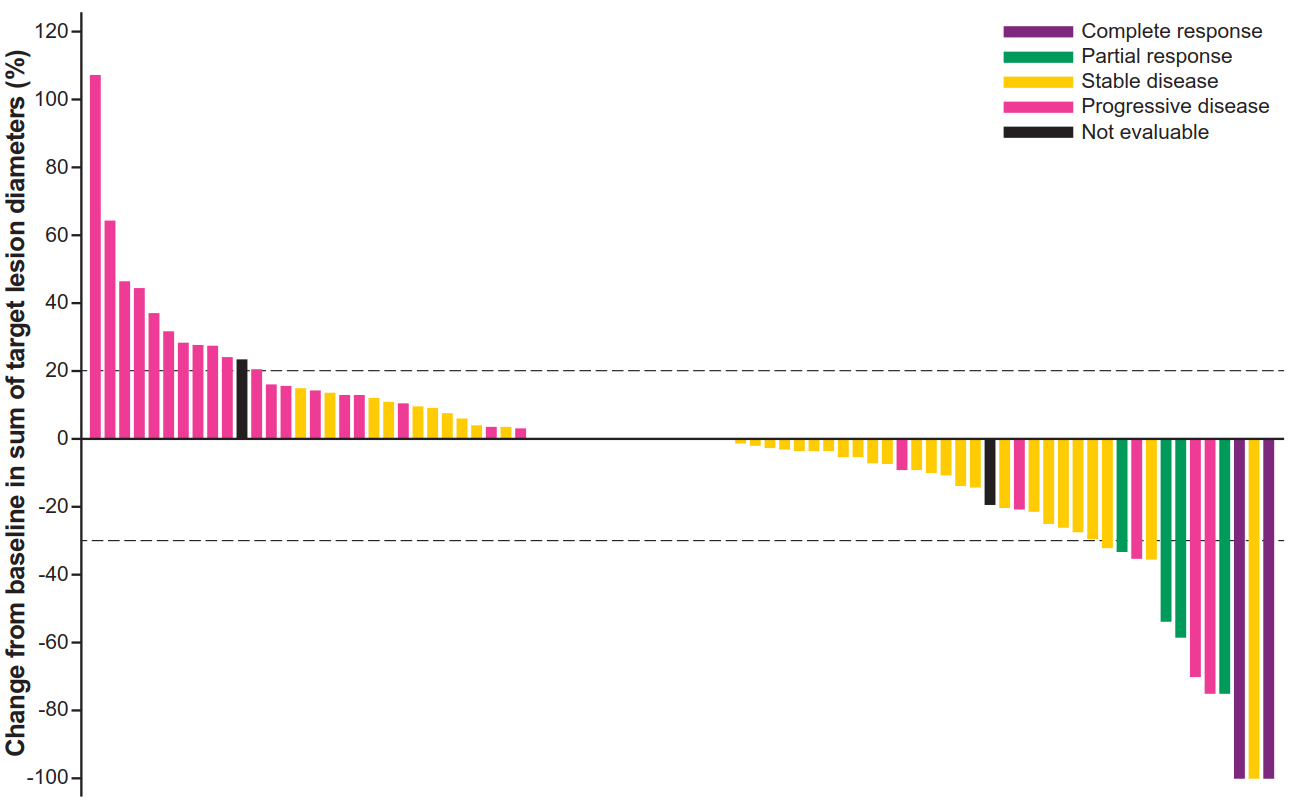


B.


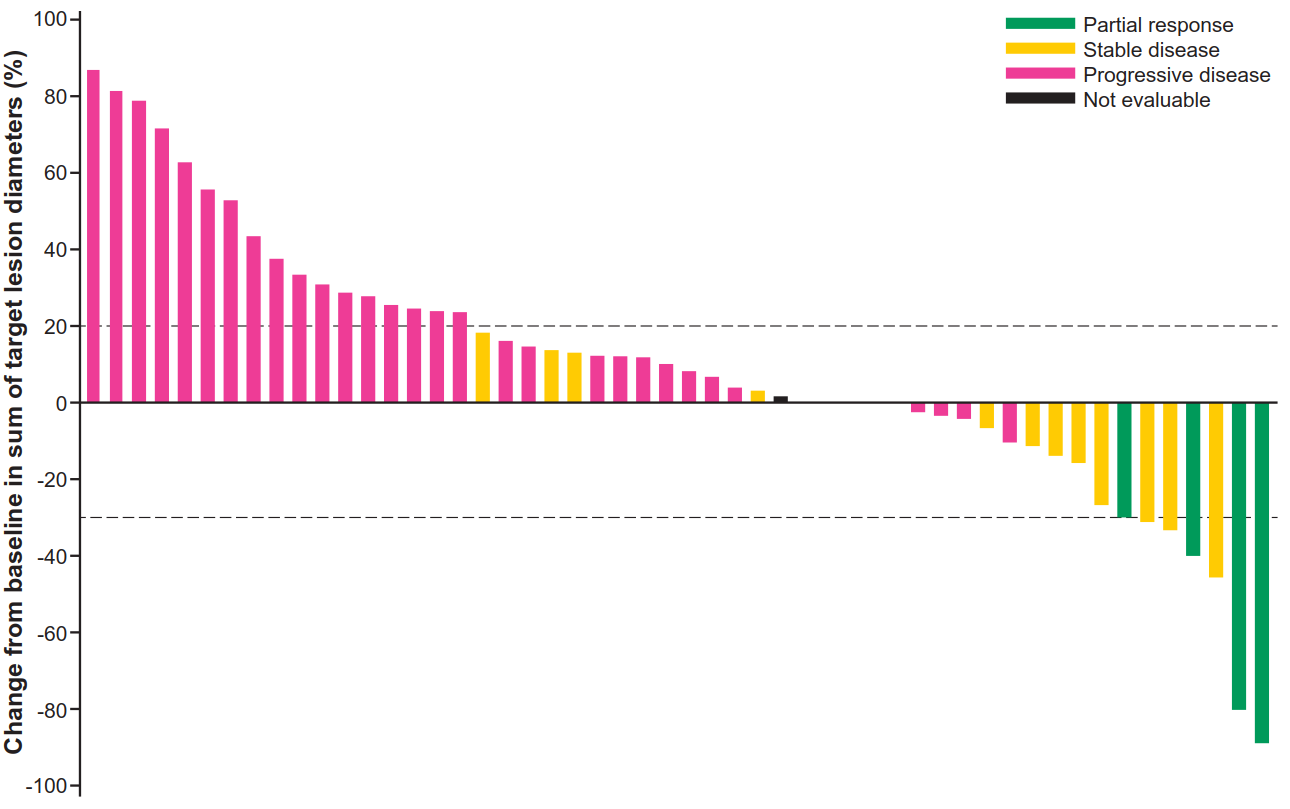


**Figure S3** Progression-free survival (PFS) by PD-L1 expression status (≥1% tumor cell cutoff) in evaluable patients. (A) First-line switch-maintenance (1L-mn) subgroup (*n*=77). (B) Second-line (2L) subgroup (*n*=45). HR, hazard ratio.

A.


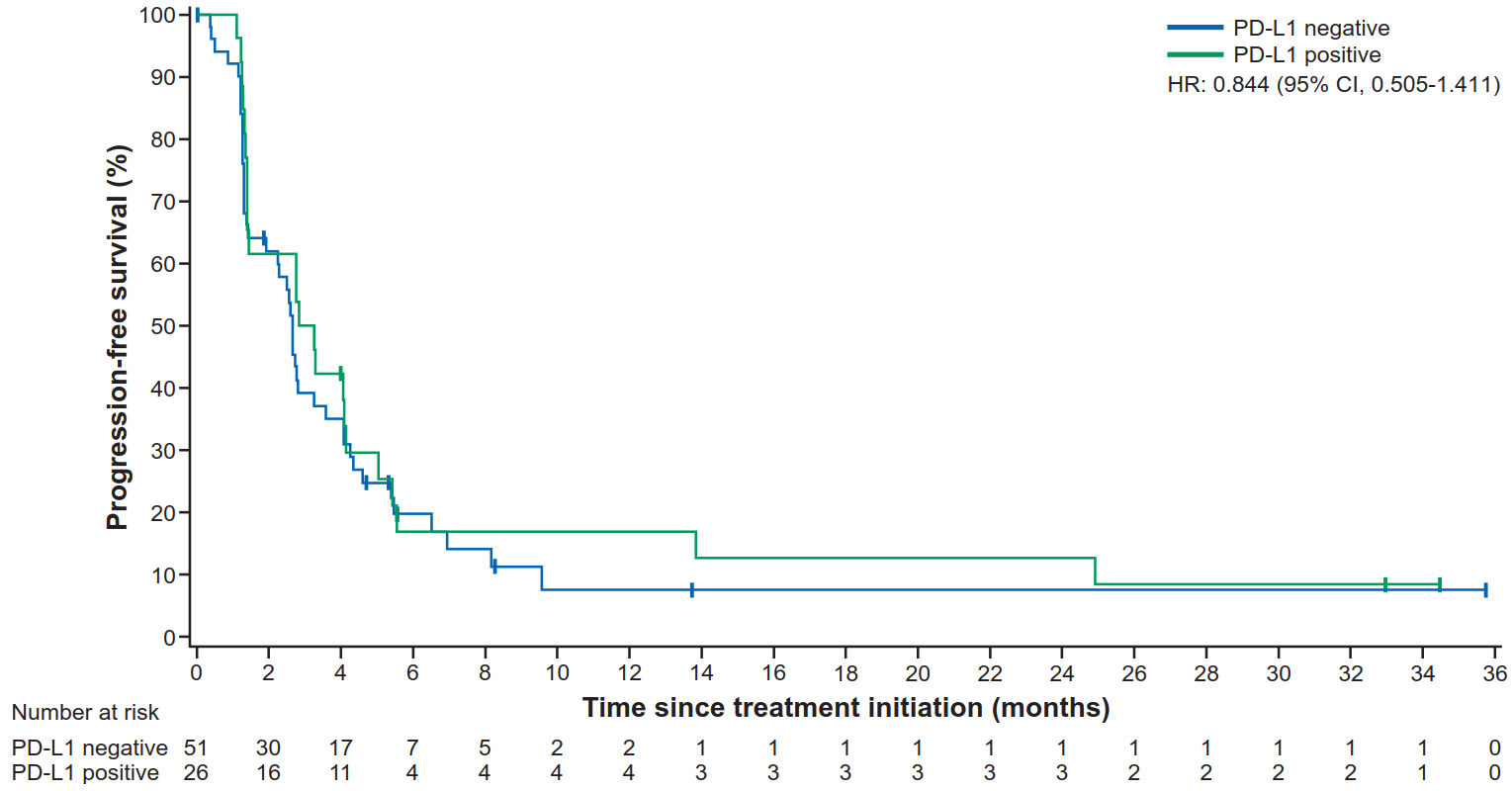


B.


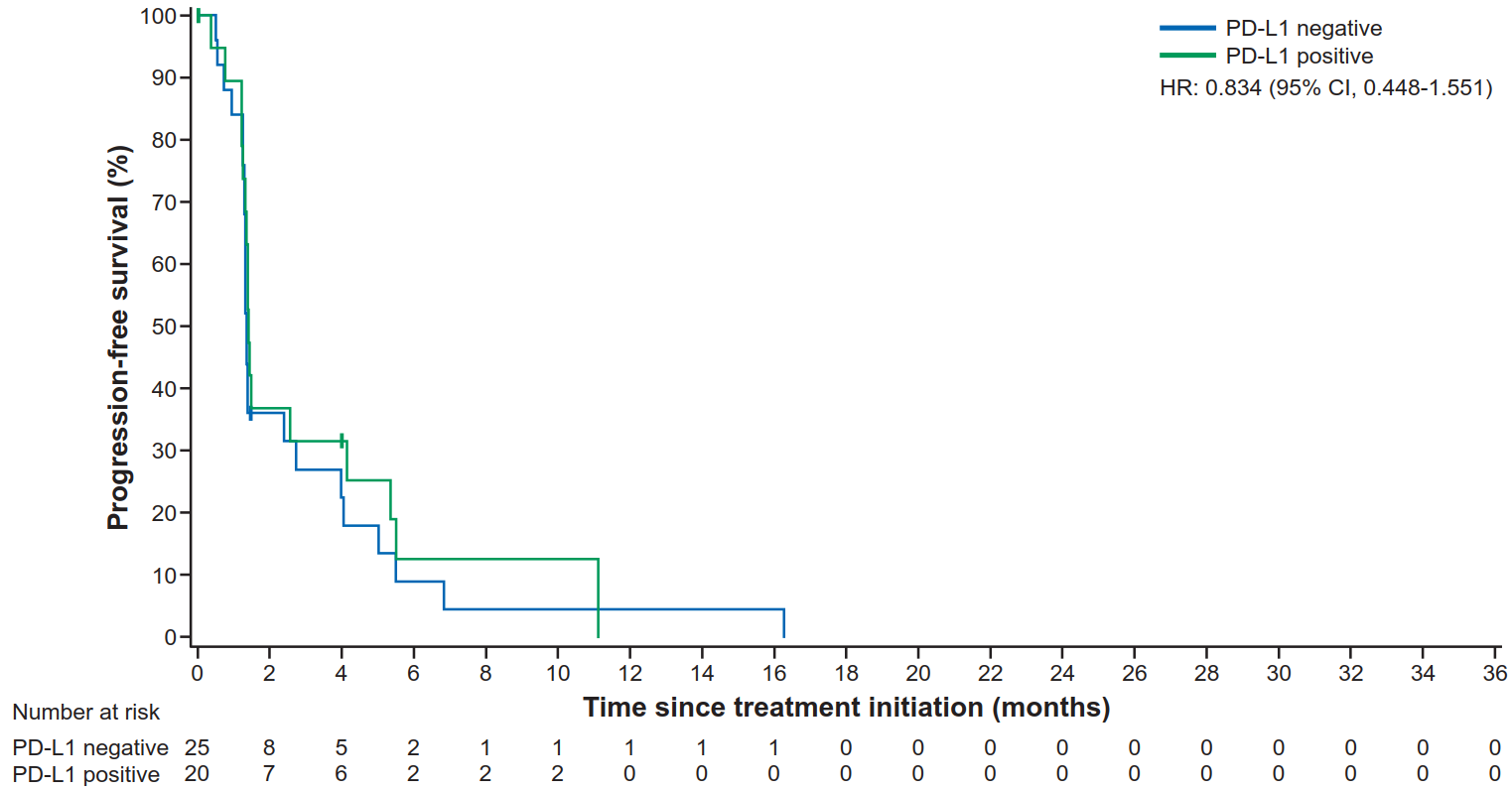

**Figure S4** Overall survival (OS) by PD-L1 expression status (≥1% tumor cell cutoff) in evaluable patients. (A) First-line switch-maintenance (1L-mn) subgroup (*n*=77). (B) Second-line (2L) subgroup (*n*=45). HR, hazard ratio.

A.


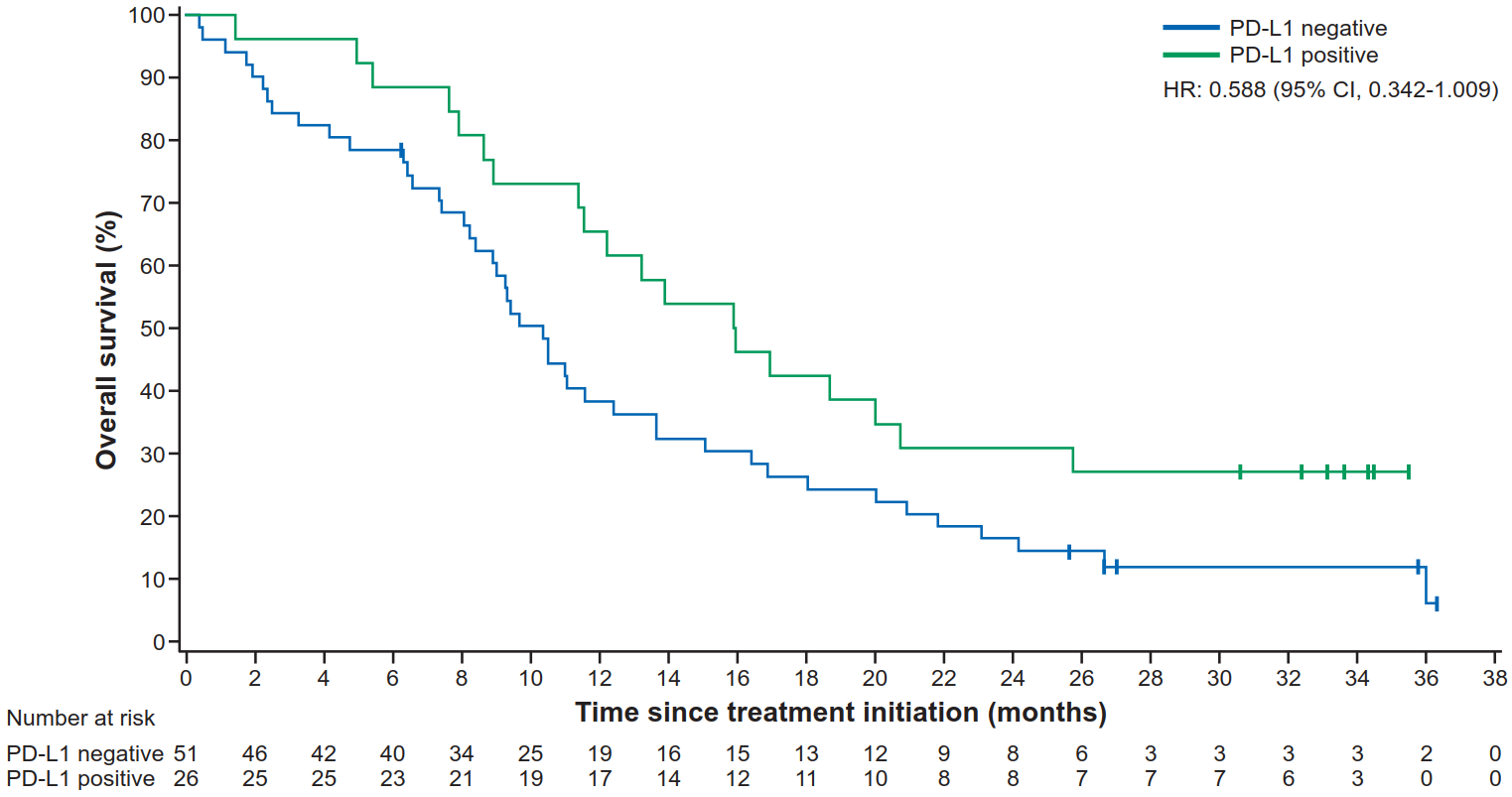


B.


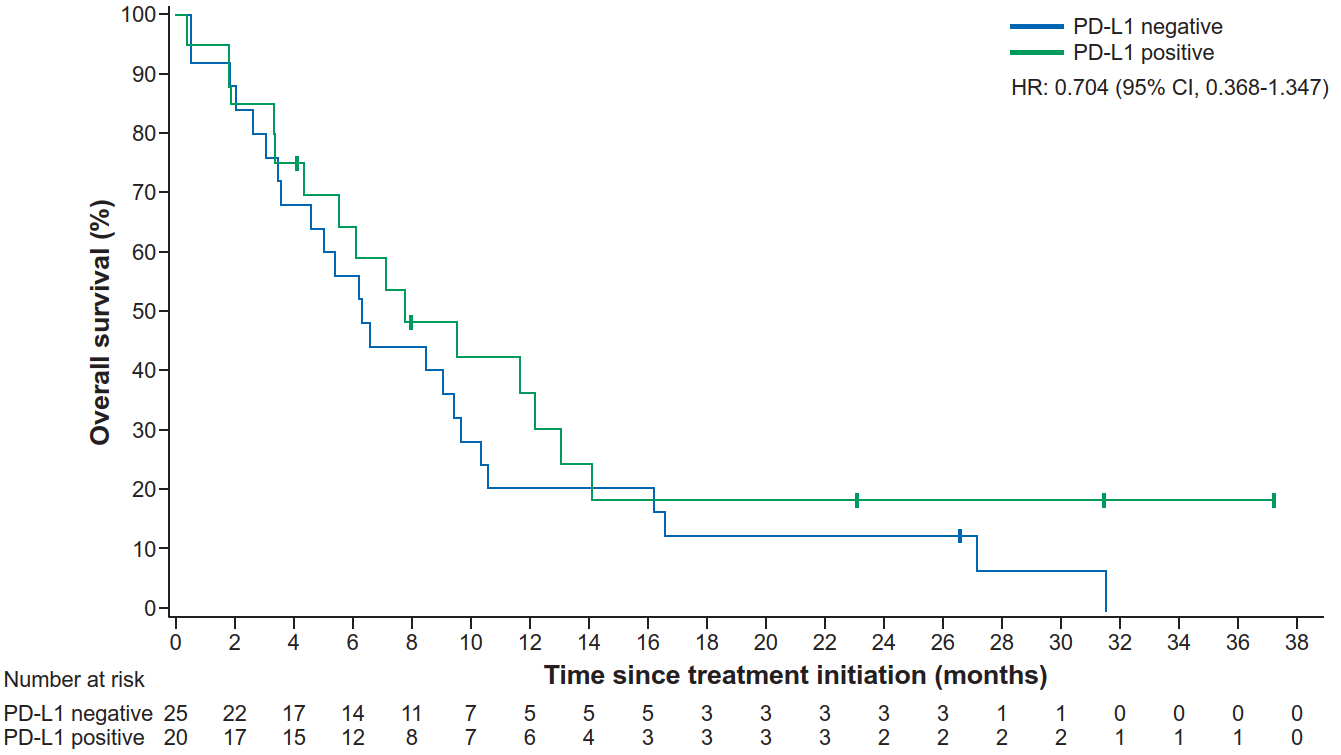

Supplement: Supplementary file 1 — Table S1. Eligibility criteria. Table S2. Response to avelumab in the 1 L-mn and 2 L subgroups. Table S3. Best response to avelumab compared with best response to prior anticancer therapy in the 1 L-mn subgroup. Table S4. Overall summary of safety. Figure S1. Time to and duration of response in responding patients. Figure S2. Best change in sum of target lesion diameters from baseline with avelumab in evaluable patients. Figure S3. Progression-free survival by PD-L1 expression status (≥1% tumor cell cutoff) in evaluable patients. Figure S4. Overall survival by PD-L1 expression status (≥1% tumor cell cutoff) in evaluable patients. (DOCX 794 kb) [file 40425_2019_508_MOESM1_ESM.docx]
